# Association between independent practice time and patient outcomes in the emergency department: a retrospective study of residents in three urban hospitals in Taiwan

**DOI:** 10.1186/s12873-023-00877-9

**Published:** 2023-09-07

**Authors:** Yi-Ying Chen, Patrick Chow-In Ko, Chien-Yu Chi, Kah Meng Chong, Yen-Pin Chen, Chien-Hua Huang

**Affiliations:** 1 Department of Emergency Medicine, National Taiwan University Hospital, National Taiwan University College of Medicine, No 7, Chung Shan S Rd (Zhongshan S Rd), Zhongzheng District, Taipei City, Taiwan; 2 Department of Emergency Medicine, National Taiwan University Hospital Yunlin Branch, Yunlin County Douliu City, Taiwan

**Keywords:** Resident autonomy, Emergency department, Supervision, Patient safety, Practice independence

## Abstract

**Background:**

The purpose of the study was to investigate the relationship between the independent practice time of residents and the quality of care provided in the Emergency Department (ED) across three urban hospitals in Taiwan. The study focused on non-pediatric and non-obstetric complaints, aiming to provide insights into the optimal balance between resident autonomy and patient safety.

**Methods:**

A comprehensive retrospective study was conducted using de-identified electronic health records (EHRs) from the hospital's integrated medical database (iMD) from August 2015 to July 2019. The independent practice time was defined as the duration from the first medical order by a resident to the first modifications by the attending physician. The primary outcome was revisits to the ED within 72 h following discharge. Statistical analysis was conducted using RStudio and pyGAM.

**Results:**

The study identified several factors associated with shorter independent practice times (< 30 minutes), including older patient age, male sex, higher body temperature, higher heart rate, lower blood pressure, and the presence of certain comorbidities. Residents practicing independently for 30–120 minutes were associated with similar adjusted odds of patient revisits to the ED (OR 1.034, 95% CI 0.978–1.093) and no higher risk of 7-day mortality (OR 0.674, 95% CI 0.592–0.767) compared to the group with less autonomy. However, independent practice times exceeding 120 minutes were associated with higher odds of revisiting the ED within 72 h. For the group with 120–210 minutes of independent practice time, the OR was 1.113 (95% CI: 1.025–1.208, *p* = 0.011). For the group with > 210 minutes, the OR was 1.259 (95% CI: 1.094–1.449, *p* = 0.001), indicating an increased risk of adverse outcomes as the independent practice time increasing.

**Conclusions:**

The study concludes that while providing residents an independent practice time between 30 to 120 minutes may be beneficial, caution should be exercised when this time exceeds 120 minutes. The findings underscore the importance of optimal supervision in enhancing patient care quality and safety. Further research is recommended to explore the long-term effects of different levels of resident autonomy on patient outcomes and the professional development of the residents themselves.

**Supplementary Information:**

The online version contains supplementary material available at 10.1186/s12873-023-00877-9.

## Introduction

Residency training is a critical phase in the professional development of physicians, shaping their clinical skills and competence for future independent practice [1]. The balance between supervision and autonomy during this period is a delicate one, with both elements playing pivotal roles. Supervision is essential to reduce medical errors and ensure patient safety [2, 3], while autonomy is crucial for residents to mature into independent practitioners [4–8]. This balance is particularly challenging to achieve in various medical specialties [6, 9, 10], including the high-pressure environment of the emergency department (ED) [11].

The ED, characterized by diverse patient encounters and time-sensitive decisions, provides a unique setting for residents to hone their skills [12–14]. However, the level of autonomy granted to residents in this setting is a complex issue. Excessive autonomy can lead to uncertainty in clinical decision-making, potentially reducing medical efficiency and compromising patient safety [9, 15]. Conversely, insufficient autonomy may impede the maturation of residents [7, 8]. Attending physicians in the ED face the intricate task of providing quality emergency care while supervising residents, a dual role that can impose stress and potentially impact their own practice efficiency [3, 13, 16, 17]. Consequently, there is considerable variability among attending physicians in the level of autonomy granted to residents [6].

As residents gain autonomy and practice independently, they face uncertainty in clinical decision-making, which can reduce medical efficiency and potentially lead to patient harm [9, 15, 18]. These concerns might be addressed by increasing accessibility to attending physician advice or by providing resident proficiency-based entrustable professional activities (EPAs) [4, 19]. In the ED, EPAs are set by illness or complaint [20, 21], but for those non-emergency residents and postgraduate year residents (PGYs) who rotate on a monthly basis, it is challenging for residents to treat patients with varying complaints and many times they may only have a single exposure to or treat a particular complaint once in the ED setting. In emergency medicine, residents are expected to have an attending physician present to provide full-time supervision at all times, and the advice of the attending physician is readily available, but supervision is sometimes too close [6]. The intensity of supervision, namely, the residency autonomy, should be adjusted according to the quality of clinical care. Although some quality and safety indicators of the ED have been documented [22], such as the length of stay (LOS), proportion of return visits to the ED [23–25], and patient mortality [26], Van Leer et al. indicated that little is known about the effect of the amount of supervision on the quality of patient care in the ED [3].

The existing literature provides valuable insights into resident autonomy and supervision in the ED, highlighting the importance of this balance for both resident development and patient safety. However, there is a paucity of evidence-based guidelines to help attending physicians determine the optimal level of autonomy for residents in the ED. Addressing this gap is crucial, as it has implications for patient outcomes, healthcare costs, and the professional growth of residents.

In light of this, our study aims to investigate the association between independent practice time and the quality of care provided by residents in the ED. We hypothesize that there is an optimal level of independent practice time that balances the benefits of resident autonomy with the need for effective supervision. By examining this association, we aim to contribute to the existing body of research on resident training in emergency medicine and provide evidence-based insights that can guide attending physicians in their supervision of residents.

To achieve this, we conducted a retrospective analysis across three urban hospitals in Taiwan, including an academic tertiary hospital and two community-based secondary hospitals. We utilized de-identified electronic health records from the hospital's integrated medical database, spanning a four-year duration. The significance of this research lies in its potential to enhance patient safety and care quality in the ED, while also promoting the professional development of residents. We recognize the challenges associated with this study, including the variability in resident experience and the diversity of patient cases, and we have taken measures to address these issues in our research design.

In conclusion, our study addresses a critical issue in residency training in the ED – the balance between resident autonomy and supervision. By investigating the association between independent practice time and the patient outcomes, we aim to provide evidence-based insights that can inform supervision practices in the ED, ultimately enhancing patient safety and care quality. This study underscores the importance of optimal supervision in enhancing patient care quality and safety, and the potential implications of insufficient autonomy or inadequate supervision. We strive to enhance the scientific background and rationale for our investigation. Ultimately, our findings may inform the development of evidence-based guidelines that optimize resident training and improve the quality of care in the ED.

## Materials and methods

### Study design and setting

This study was approved by National Taiwan University Hospital Research Ethics Committee (201902078RINB; 202107133RINA). We conducted a retrospective study of three urban hospitals, including the ED of an academic tertiary hospital in Taipei city with approximately 100,000 annual visits, and the EDs of two community-based secondary hospitals in Hsinchu city and Yulin county with approximately 60,000 visits each. In the academic ED, there are typically 12 attending physicians who work with 26 residents and PGYs on a daily basis. In contrast, in a community ED, residents and PGYs are not regular monthly staff, and the ED patients are typically seen by approximately six shiftwork attending physicians on a daily basis. Attending physicians work 12-h shifts and then switch to the next shift; residents and PGYs work 8-h or 12-h shifts and hand patients over to the attending physician. For those patients initially evaluated by a resident physician or PGY, treatment planning and disposition are supervised by the attending physician in charge.

**Fig. 1:**
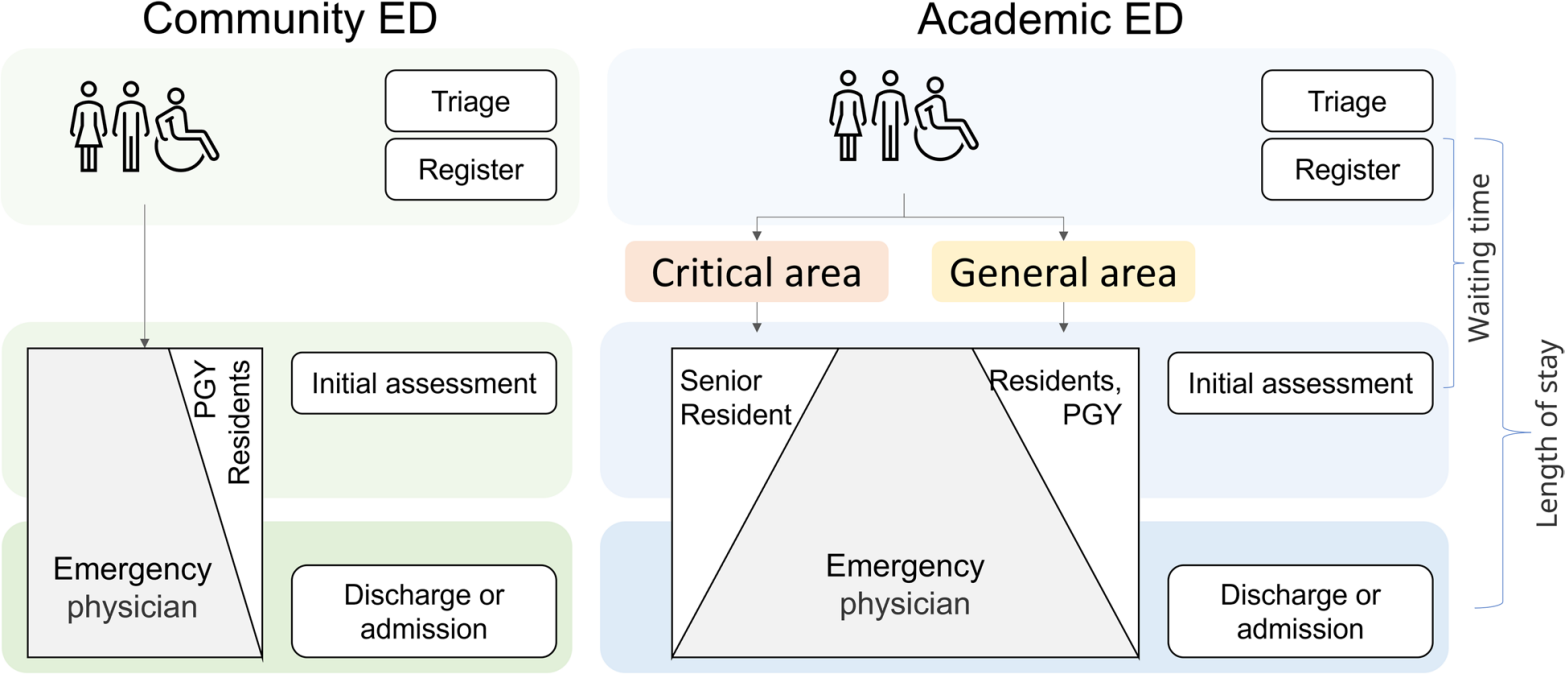
ED patient visit process of community and academic hospitals. This figure showcases the stages involved in a patient's visit, such as triage, registration, assessment, and discharge. The main difference between the two types of EDs is the presence of a dedicated critical care area in academic EDs that is staffed by senior residents and attending physicians to provide prompt and efficient care. The time elapsed between triage/registration and the first assessment by a physician is known as the waiting time, while the time elapsed between triage/registration and the patient's departure from the ED is referred to as the LOS

Each ED patient is triaged according to the emergency severity level and randomly assigned to an attending physician. The community EDs have only one area for patients, while in the academic ED, the patients are pre-assigned to general or critical areas based on their conditions, and the attending physicians who are responsible for a particular area evaluate and manage the patient accordingly (Fig. 1). The critical area is a dedicated intensive care area of the academic ED that provides expedited clinical services. It is staffed by attending physicians and senior emergency medicine residents specifically trained to care for patients requiring urgent treatment or resuscitation. These patients typically have severe conditions such as severe hypotension, tachy- or bradycardia, respiratory failure, myocardial ischemia, or stroke within a specific time window, which make them candidates for the critical area. The general area is staffed by attending physicians, PGYs, and emergency medicine, family medicine, and internal medicine residents. Except for the attending physician and ED residents, all other doctors rotate in the ED for one month. The academic year begins in August, with each PGY and resident earning a new title and starting the training process for the year.

### Data collection

In this study, we utilized de-identified electronic health records (EHRs) from the hospital's integrated medical database (iMD) spanning from August 2015 to July 2019, in which all records pertaining to patients or staff were anonymized, with only employee titles and seniority preserved. The requirement for consent was waived by the ethics committee due to the de-identified nature of the data.

The selection of this specific timeframe was underpinned by several considerations. Primarily, this period precedes the onset of the COVID-19 pandemic, which necessitated the implementation of stringent infection control measures that substantially altered patient management protocols, patient demographics, and the frequency of modifications in clinical procedures. These alterations could potentially introduce confounding variables into our study. Secondly, the commencement of our study period in 2015 corresponds with the five-year mark post the implementation of the EHR system within the hospital network. This ensured the availability of comprehensive and stable medical data. Moreover, during this period, the duration of the emergency medicine residency program was consistently 42 months. Prior to 2015, the training program had a different duration of 48 months, introducing an additional variable that could impact our study results. Lastly, the academic year in our institution commences in August, providing a logical basis for the selected start and end points of our study period.

Our patient population was derived from all patient visits registered in the iMD during the study period. We specifically targeted patients who accessed the hospital's healthcare resources through the emergency department, as indicated by a specific flag in their records. This flag served as our primary inclusion criterion, allowing us to focus on the population of interest. From this initial pool of patients, we applied further selection criteria to refine our study group. We included patients who were assigned to areas within the emergency department that were served by emergency physicians. This was determined based on the area assignment in their EHRs upon their arrival at the emergency department.

We focused on patients presenting with non-pediatric and non-obstetric complaints, with one notable exception. Children presenting with injuries or trauma were included in our study, as these patients would typically be assigned to areas served by emergency physicians rather than pediatricians. Patients who were assigned to areas primarily cared for or seen by pediatric or obstetric doctors, with the exception of those children presenting with injuries or trauma, were excluded from our study. These criteria were chosen to align with the scope of practice of the resident physicians in our study, who primarily handle adult, non-obstetric emergencies, and pediatric traumatic emergencies.

For patients with multiple visits within a 24-h period, we only included the first record in our analysis. We then applied several exclusion criteria to further refine our study population. Firstly, we excluded patients who experienced out-of-hospital cardiac arrests (OHCAs) or those who were assigned to the critical area. This decision was made because the management of cardiac arrest patients could conflict with the mortality outcomes we intended to observe in our study. Furthermore, our study aimed to determine an acceptable level of independent practice time for young doctors, which is a different context than the immediate and intensive medical intervention required for cardiac arrest patients. Secondly, we excluded patients who were initially seen by an attending physician. For each patient, we arranged all medical orders chronologically and identified the first order created by an attending physician. We utilized a binary variable to denote whether the first medical order was issued by an attending physician, which served as our exclusion criterion.

Lastly, we excluded patients with typos in their vital signs on the triage sheet. In this study, typos were defined as instances where the diastolic blood pressure was recorded as higher than the systolic blood pressure, systolic blood pressure exceeded 300 mmHg, heart rate was over 250 beats per minute, respiratory rate was above 50 breaths per minute, body temperature was recorded as higher than 48°C or lower than 10°C, and weight was recorded as over 400 kg. The rationale for excluding these typos is multifaceted. Firstly, these errors, likely due to human error during data entry, can significantly skew the data, especially as they often exceed the standard deviation by more than tenfold. This could potentially distort our analysis and lead to inaccurate results. Secondly, these typos are random occurrences, as our previous studies examined the nature and frequency of these data entry errors [26, 27]. Thirdly, given the large volume of data in our study, the exclusion of these random typos might not significantly impact the overall findings. Therefore, to maintain the integrity and reliability of our analysis, we opted to discard these anomalous data points.

### Variables and outcome measurements

We collected the patient characteristics, first vital signs in the ED, comorbidities, chief complaints, emergency severity index, disposition, clinical time-stamped information, treatments, examinations, and final medical expenses. The characteristics included age, sex, and body weight; the vital signs included temperature, heart rate, respiratory rate, oxygen saturation, blood pressure, and levels of consciousness evaluated using the Glasgow Coma Scale (GCS). During the data collection process, we encountered instances of missing data due to the unavailability of certain variables for some patients. To address this challenge and maintain the integrity of our dataset, we employed the Expectation–Maximization algorithm [27, 28]. This statistical technique allowed us to estimate probable values for these missing data points based on the other available data, thereby ensuring a robust and complete dataset for our subsequent analyses. This approach upheld the integrity of our data, ensuring that our subsequent analyses and findings were based on a comprehensive and representative sample of our study cohort.

We selected comorbidities based on the Charlson Comorbidity Index (CCI) [29], including coronary artery disease, heart failure, hypertension, diabetes mellitus, chronic obstructive pulmonary disease (COPD), stroke, end-stage renal disease, liver cirrhosis, peptic ulcer disease, peripheral vascular disease, cancer, and rheumatic disease. To ensure the accuracy of our comorbidity identification process, we adopted an approach using patient medical record data. We focused on patient diagnostic history and specifically examined records up until the triage date, considering only relevant and valid entries. The coding process involved scanning the diagnostic text for key phrases related to each disease, relying on a variety of terms and abbreviations specific to each comorbidity to maximize detection accuracy. For instance, to identify patients with coronary artery disease, we searched for phrases like 'coronary artery disease', 'POBAS', 'CAD', and 'ischemic heart'. Similar techniques were used for other conditions, using corresponding disease-specific terminologies. In situations where no valid diagnostic history was found, we assigned a default value of zero to the respective comorbidity category.

We defined the waiting time as the time interval between the completion of registration in the ED and the start of the diagnostic evaluation. The chief complaints were divided into two groups: trauma and non-trauma. Treatments and examinations were grouped by the prescriber's seniority and ordered chronologically. The time to first supervision was defined as the interval from the first plan of a resident or PGY to the first modifications or documentation by the attending physician; this was divided into independent practice time groups defined in 30-min increments. In this study, close supervision was defined as the physician initiating supervision within 30 min and was considered to be indicative of less resident autonomy due to independent practice < 30 min. In addition, the lack of modification or documentation by the attending physician before a patient's discharge from the ED was considered non-recorded.

The primary outcome of our study is centered on revisits to the ED within 72 h following the last ED discharge [23]. An ED revisit is defined as a return to the ED that results in a new, separate encounter within the 72-h post-discharge window, irrespective of the complaint or condition triggering the revisit. Our secondary outcomes encompass several elements. The first one is mortality within seven days following the ED visit [26]. Here, mortality refers to any death occurrence within seven days from the initial ED visit, regardless of the cause or location of death. Another secondary outcome is the LOS in the ED, defined as the duration of the patient's stay within the ED department, measured in minutes. The LOS is calculated as the time interval from the patient's recorded arrival time until the official departure from the ED. Departure could occur due to several reasons such as discharge, admission to the hospital, or transfer to another healthcare facility. Finally, the total medical expenses incurred during the ED visit constitute our last secondary outcome. These expenses encapsulate all the costs associated with the patient's care during their stay in the ED, inclusive of diagnostics, therapeutic procedures, medication, and facility charges.

### Statistical analysis

Continuous variables are summarized as means with standard deviations (SDs) or medians with interquartile ranges (IQRs) and were examined using the one-way ANOVA and the Kruskal–Wallis test. Categorical variables are presented in case numbers and proportions and were analyzed using the Chi-square test. We applied a multivariable logistic regression model to investigate adjusted associations between independent practice time groups and binary outcomes by controlling for variables with statistical significance in the univariable regression analysis. Except for the reference group, adjacent independent practice time groups with similar odds ratios (ORs) for the primary outcome were merged into a supergroup. This approach was adopted to reduce the number of groups and to coherently analyze clusters of adjacent categories that demonstrated similar performance characteristics. We also adopted a multivariable linear regression model to examine adjusted associations between independent practice time groups and continuous outcomes. All ORs and beta-coefficients are presented with 95% confidence intervals (CIs). In this study, we also adopted a generalized additive model (GAM) to demonstrate the adjusted associations between variables and outcomes.

In this study, we subjected continuous variables to standardization, a process that transformed each variable to have a mean of 0 and a standard deviation of 1. This procedure not only enables a more direct comparison of the importance of different predictors in the model but also facilitates the interpretation of the model's results [30]. The statistical analysis was conducted using RStudio (version 2022.07.1 + 554) based on R (version 4.2.1) and pyGAM (version 0.8.0) based on python (version 3.8.11). All p-values in this study were two-sided and were considered statistically significant when less than 0.05. To ensure the robustness of our statistical results, we performed a power analysis using the simulation method [31, 32].

## Results

**Fig. 2:**
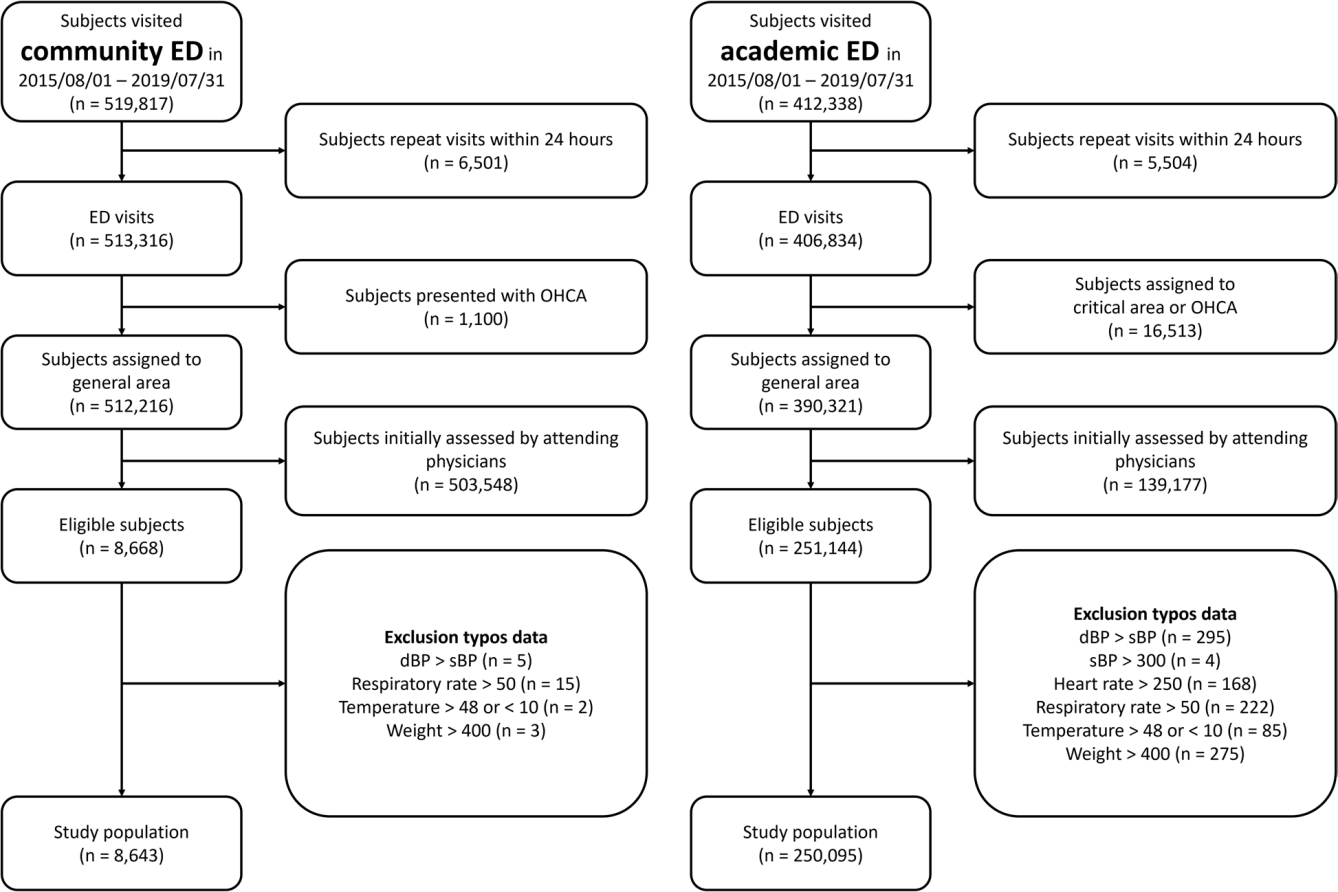
Study flow diagram. Our study began with all patient visits registered in the iMD from August 2015 to July 2019. We targeted patients accessing healthcare via the emergency department. For multiple visits within 24 hours, only the first record was analyzed. We excluded patients with out-of-hospital cardiac arrests, those initially seen by an attending physician, and those with typos in their vital signs

During the 4-year study period, a total of 932,155 non-pediatric and non-obstetric emergency visits were recorded, of which 920,150 were unique visits (Fig. 2). The majority of visits in the academic ED (96%) were assigned to the general area, and 36% of these visits were seen directly by attending physicians. In contrast, in community EDs, 98% of patients were seen directly by attending physicians. There were some errors in the data, with typos occurring in 0.4% of visits, but these visits were excluded from the analysis, leaving 258,738 patient visits for analysis.

### Characteristics of the study population

**Fig. 3:**
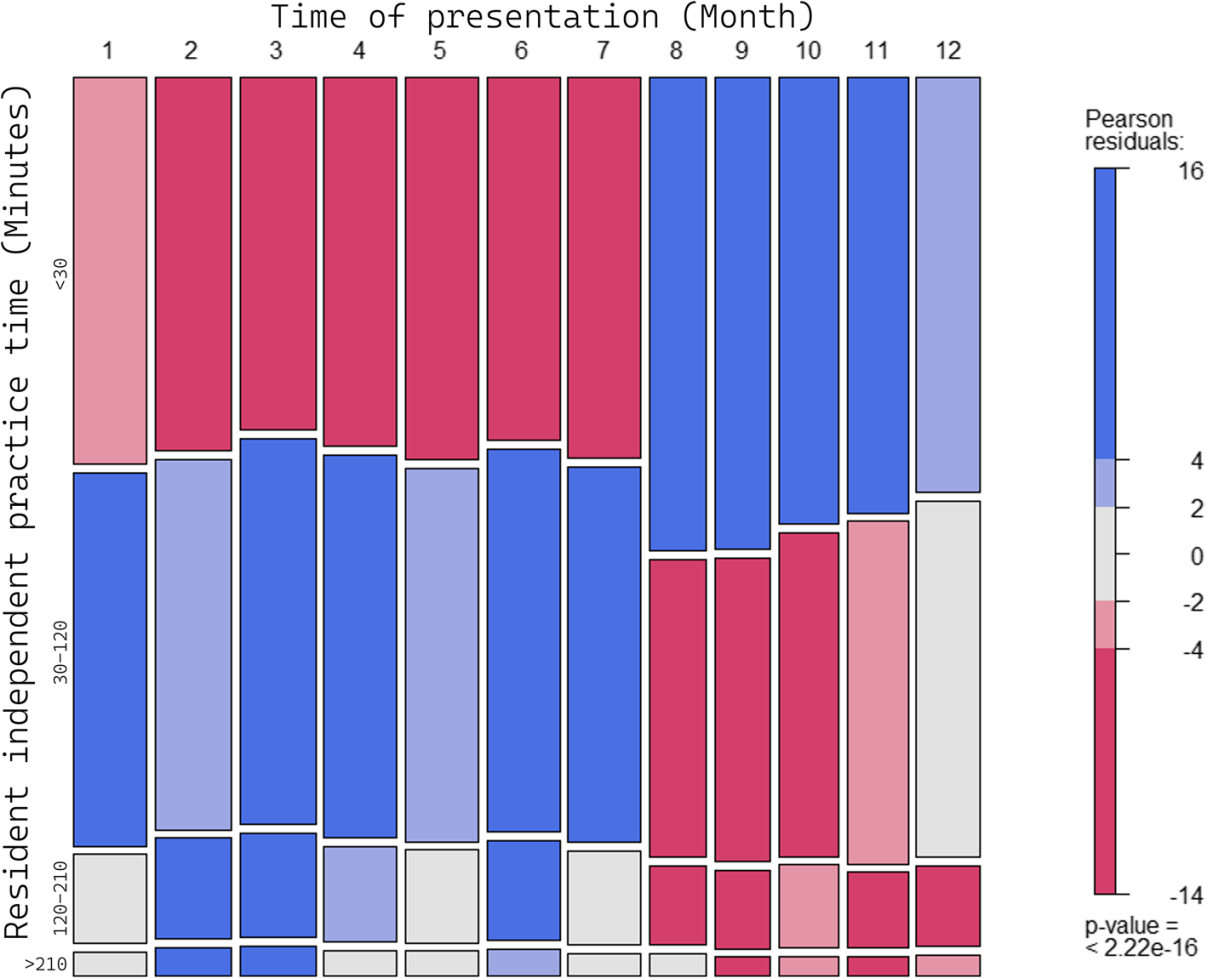
Mosaic plot showing the results of a chi-square analysis exploring the correlation between the independent practice time group (as categorized by the number of minutes) and the month in which the patient visited the ED. It displays the standardized residuals of this analysis

The majority of the study cohort was comprised of non-trauma patients, representing 85% (220,657 of 258,738) of the total patient population. These non-trauma patients were typically older (mean age of 55 years), predominantly female (53.1% compared to 48.1% in trauma patients), exhibited a higher prevalence of comorbidities (35.6% versus 13.8%), and had a higher likelihood of hospitalization (18.8% versus 11.1%) relative to their trauma counterparts. Detailed demographic and clinical characteristics are delineated in Table 1. The subgroup of discharged patients had a mean age of 50 years, with males constituting 46% of this population. Four percent of this subgroup did not have a determined independent practice time as there was a lack of modification or documentation by the attending physician before their discharge from the ED (Table 2). Eighty-three percent of patients presented to a resident or PGY practicing independently for < 120 min, and less than half presented to a resident practicing independently for < 30 min. In addition, patients with some emergency severity indices (1 and 5) tended to receive closer supervision by an attending physician, with odds ratios of 1.393 (1.128–1.721, *p* = 0.002) and 3.187 (2.914–3.486, *p* < 0.001) respectively. The percentage of close supervision also increased during the months of August, September, October, November, and December with odds ratios ranging from 1.146 (1.102–1.192, *p* < 0.001) in December to 1.513 (1.453–1.576, *p* < 0.001) in August. This uptick in close supervision may be attributable to the influx of new medical personnel at the commencement of the academic year, among other contributing factors (Fig. 3). Furthermore, the study identified several patient characteristics and vital signs associated with close supervision. For instance, male patients were more likely to be closely supervised (OR = 1.193, *p* < 0.001), as were patients with higher heart rates (OR = 1.096, *p* < 0.001) and respiratory rates (OR = 1.111, *p* < 0.001). Conversely, patients with higher oxygen saturation and blood pressure were less likely to be closely supervised (OR = 0.951 and 0.967 respectively, *p* < 0.001 for both) (Table 3).

**Table 1:** Baseline clinical characteristics of the included patients

Variable	All (*n* = 258,738)	Academic (*n* = 250,095)	Community (*n* = 8,643)	*p*-value	Non-trauma (*n* = 220,657)	Trauma (*n* = 38,081)	*p*-value
Age, mean (SD), year	52.6 (21.7)	52.6 (21.7)	54.1 (22.0)	< .001	54.9 (20.0)	39.6 (26.2)	< .001
Male, *n* (%)	123,343 (47.7)	119,004 (47.6)	4,339 (50.2)	< .001	103,568 (46.9)	19,775 (51.9)	< .001
Vital sign							
Body temperature, mean (SD), °C	36.9 (1.1)	37.0 (1.1)	36.9 (1.5)	< .001	37.0 (1.1)	36.8 (1.2)	< .001
Heart rate, mean (SD), beats per min	90.5 (19.5)	90.6 (19.4)	89.5 (20.8)	< .001	90.7 (19.1)	89.7 (21.6)	< .001
Respiratory rate, mean (SD), breaths per min	19.3 (2.2)	19.3 (2.2)	19.0 (2.7)	< .001	19.2 (2.0)	19.6 (3.1)	< .001
Oxygen saturation, median (IQR), %	98.0 (3.0)	98.0 (3.0)	98.0 (3.0)	< .001	97.0 (3.0)	98.0 (3.0)	< .001
Systolic blood pressure, mean (SD), mmHg	135.4 (27.4)	135.2 (27.2)	140.3 (30.5)	< .001	135.8 (27.0)	133.3 (29.2)	< .001
Diastolic blood pressure, mean (SD), mmHg	77.3 (15.0)	77.3 (14.9)	78.5 (17.4)	< .001	77.3 (14.7)	77.3 (16.7)	0.282
GCS-E, *n* (%)				< .001			< .001
1	284 (0.1)	216 (0.1)	68 (0.8)		262 (0.1)	22 (0.1)	
2	796 (0.3)	736 (0.3)	60 (0.7)		778 (0.4)	18 (0.0)	
3	1,662 (0.6)	1,594 (0.6)	68 (0.8)		1,582 (0.7)	80 (0.2)	
4	255,996 (98.9)	247,549 (99.0)	8,447 (97.7)		218,035 (98.8)	37,961 (99.7)	
GCS-V, *n* (%)				< .001			< .001
1	1,819 (0.7)	1,647 (0.7)	172 (2.0)		1,749 (0.8)	70 (0.2)	
2	2,130 (0.8)	1,996 (0.8)	134 (1.6)		2,053 (0.9)	77 (0.2)	
3	1,014 (0.4)	973 (0.4)	41 (0.5)		948 (0.4)	66 (0.2)	
4	2,094 (0.8)	2,038 (0.8)	56 (0.6)		1,873 (0.8)	221 (0.6)	
5	251,681 (97.3)	243,441 (97.3)	8,240 (95.3)		214,034 (97.0)	37,647 (98.9)	
GCS-M, *n* (%)				< .001			< .001
1	148 (0.1)	110 (0.0)	38 (0.4)		139 (0.1)	9 (0.0)	
2	103 (0.0)	87 (0.0)	16 (0.2)		97 (0.0)	6 (0.0)	
3	335 (0.1)	268 (0.1)	67 (0.8)		321 (0.1)	14 (0.0)	
4	1,593 (0.6)	1,467 (0.6)	126 (1.5)		1,551 (0.7)	42 (0.1)	
5	3,667 (1.4)	3,538 (1.4)	129 (1.5)		3,468 (1.6)	199 (0.5)	
6	252,892 (97.7)	244,625 (97.8)	8,267 (95.6)		215,081 (97.5)	37,811 (99.3)	
Body weight, mean (SD), kg	58.5 (19.8)	59.5 (18.3)	29.1 (32.5)	< .001	59.8 (18.4)	51.1 (25.1)	< .001
Comorbidity	83,719 (32.4)	81,703 (32.7)	2,016 (23.3)	< .001	78,466 (35.6)	5,253 (13.8)	< .001
Coronary artery disease	14,260 (5.5)	13,938 (5.6)	322 (3.7)	< .001	13,511 (6.1)	749 (2.0)	< .001
Malignancy	38,367 (14.8)	37,826 (15.1)	541 (6.3)	< .001	36,938 (16.7)	1,429 (3.8)	< .001
Heart failure	9,016 (3.5)	8,765 (3.5)	251 (2.9)	0.003	8,535 (3.9)	481 (1.3)	< .001
COPD	4,321 (1.7)	4,115 (1.6)	206 (2.4)	< .001	4,130 (1.9)	191 (0.5)	< .001
Stroke	21,765 (8.4)	21,274 (8.5)	491 (5.7)	< .001	20,548 (9.3)	1,217 (3.2)	< .001
Dementia	3,636 (1.4)	3,502 (1.4)	134 (1.6)	0.244	3,388 (1.5)	248 (0.7)	< .001
Diabetes mellitus	24,274 (9.4)	23,516 (9.4)	758 (8.8)	0.047	22,856 (10.4)	1,418 (3.7)	< .001
End-stage renal disease	6,307 (2.4)	6,133 (2.5)	174 (2.0)	0.009	6,022 (2.7)	285 (0.7)	< .001
Hypertension	63,651 (24.6)	62,066 (24.8)	1,585 (18.3)	< .001	59,347 (26.9)	4,304 (11.3)	< .001
Liver cirrhosis	5,775 (2.2)	5,610 (2.2)	165 (1.9)	0.039	5,562 (2.5)	213 (0.6)	< .001
Peptic ulcer disease	2,544 (1.0)	2,475 (1.0)	69 (0.8)	0.076	2,414 (1.1)	130 (0.3)	< .001
Peripheral vascular disease	1,965 (0.8)	1,908 (0.8)	57 (0.7)	0.276	1,854 (0.8)	111 (0.3)	< .001
Rheumatic disease	6,512 (2.5)	6,392 (2.6)	120 (1.4)	< .001	6,107 (2.8)	405 (1.1)	< .001
Time of presentation							
Month, *n* (%)				< .001			< .001
1	23,518 (9.1)	22,353 (8.9)	1,165 (13.5)		20,073 (9.1)	3,445 (9.0)	
2	23,656 (9.1)	22,919 (9.2)	737 (8.5)		20,319 (9.2)	3,337 (8.8)	
3	23,373 (9.0)	22,381 (8.9)	992 (11.5)		20,011 (9.1)	3,362 (8.8)	
4	22,203 (8.6)	21,561 (8.6)	642 (7.4)		19,092 (8.7)	3,111 (8.2)	
5	22,808 (8.8)	21,817 (8.7)	991 (11.5)		19,352 (8.8)	3,456 (9.1)	
6	22,321 (8.6)	21,360 (8.5)	961 (11.1)		19,005 (8.6)	3,316 (8.7)	
7	22,163 (8.6)	21,893 (8.8)	270 (3.1)		19,083 (8.6)	3,080 (8.1)	
8	19,224 (7.4)	18,962 (7.6)	262 (3.0)		16,510 (7.5)	2,714 (7.1)	
9	18,328 (7.1)	17,963 (7.2)	365 (4.2)		15,668 (7.1)	2,660 (7.0)	
10	19,579 (7.6)	18,970 (7.6)	609 (7.0)		16,719 (7.6)	2,860 (7.5)	
11	20,091 (7.8)	19,120 (7.6)	971 (11.2)		16,802 (7.6)	3,289 (8.6)	
12	21,474 (8.3)	20,796 (8.3)	678 (7.8)		18,023 (8.2)	3,451 (9.1)	
Day of the week, *n* (%)				< .001			< .001
Sunday	42,211 (16.3)	40,906 (16.4)	1,305 (15.1)		35,899 (16.3)	6,312 (16.6)	
Monday	38,276 (14.8)	36,925 (14.8)	1,351 (15.6)		32,769 (14.9)	5,507 (14.5)	
Tuesday	36,886 (14.3)	35,506 (14.2)	1,380 (16.0)		31,577 (14.3)	5,309 (13.9)	
Wednesday	32,905 (12.7)	31,757 (12.7)	1,148 (13.3)		28,090 (12.7)	4,815 (12.6)	
Thursday	34,037 (13.2)	32,920 (13.2)	1,117 (12.9)		29,098 (13.2)	4,939 (13.0)	
Friday	35,485 (13.7)	34,387 (13.7)	1,098 (12.7)		30,286 (13.7)	5,199 (13.7)	
Saturday	38,938 (15.0)	37,694 (15.1)	1,244 (14.4)		32,938 (14.9)	6,000 (15.8)	
Time of the day, *n* (%)				< .001			< .001
00:00–04:00	27,179 (10.5)	26,682 (10.7)	497 (5.8)		24,805 (11.2)	2,374 (6.2)	
04:00–08:00	21,960 (8.5)	21,565 (8.6)	395 (4.6)		20,313 (9.2)	1,647 (4.3)	
08:00–12:00	48,312 (18.7)	46,052 (18.4)	2,260 (26.1)		42,477 (19.3)	5,835 (15.3)	
12:00–16:00	52,702 (20.4)	50,616 (20.2)	2,086 (24.1)		44,132 (20.0)	8,570 (22.5)	
16:00–20:00	55,615 (21.5)	53,497 (21.4)	2,118 (24.5)		44,723 (20.3)	10,892 (28.6)	
20:00–24:00	52,970 (20.5)	51,683 (20.7)	1,287 (14.9)		44,207 (20.0)	8,763 (23.0)	
Triage level, *n* (%)				< .001			< .001
1	528 (0.2)	337 (0.1)	191 (2.2)		476 (0.2)	52 (0.1)	
2	44,505 (17.2)	43,532 (17.4)	973 (11.3)		40,470 (18.3)	4,035 (10.6)	
3	194,476 (75.2)	187,889 (75.1)	6,587 (76.2)		166,266 (75.4)	28,210 (74.1)	
4	16,642 (6.4)	15,817 (6.3)	825 (9.5)		11,458 (5.2)	5,184 (13.6)	
5	2,587 (1.0)	2,520 (1.0)	67 (0.8)		1,987 (0.9)	600 (1.6)	
Waiting time, median (IQR), minutes	6.7 (10.9)	6.8 (11.1)	4.4 (5.6)	< .001	7.0 (11.6)	5.1 (7.6)	< .001
Time to first supervision, median (IQR), minutes	35.0 (73.0)	36.2 (74.3)	19.9 (33.0)	< .001	38.5 (77.2)	24.7 (45.7)	< .001
Disposition				< .001			< .001
Discharge, *n* (%)	203,864 (78.8)	197,337 (78.9)	6,527 (75.5)		170,571 (77.3)	33,293 (87.4)	
Admission, *n* (%)	45,709 (17.7)	43,934 (17.6)	1,775 (20.5)		41,491 (18.8)	4,218 (11.1)	
Outcomes							
Revisit within 72 h of last discharge, *n* (%)	7,472 (2.9)	7,123 (2.8)	349 (4.0)	< .001	6,903 (3.1)	569 (1.5)	< .001
Mortality within 7 days after the ED visit, *n* (%)	1,305 (0.5)	1,268 (0.5)	37 (0.4)	0.309	1,272 (0.6)	33 (0.1)	< .001
LOS, median (IQR), minutes	162.1 (438.7)	162.8 (455.0)	141.4 (241.2)	< .001	184.2 (669.1)	77.2 (99.0)	< .001
Medical expenses, median (IQR), dollar	2,885.0 (5,030.0)	2,880.0 (5,049.0)	3,013.0 (4,525.5)	0.018	3,044.0 (5,636.0)	1,986.0 (2,646.0)	< .001

**Table 2:** Discharged patient characteristics and clinical outcomes by the supervision group

Variable	Resident independent practice time (minutes)						
	All discharged subjects (*n* = 203,864)	< 30 (*n* = 84,709)	30 to 120 (*n* = 83,861)	120 to 210 (*n* = 21,483)	> 210 (*n* = 5,322)	Non-recorded (*n* = 8,489)	*p* value
Age, mean (SD), year	50.2 (21.7)	48.9 (22.8)	50.8 (20.8)	53.9 (19.9)	56.5 (19.4)	44.9 (21.4)	< .001
Male, *n* (%)	94,000 (46.1)	40,986 (48.4)	37,470 (44.7)	9,238 (43.0)	2,356 (44.3)	3,950 (46.5)	< .001
Vital sign							
Body temperature, mean (SD), °C	36.9 (1.0)	36.9 (1.0)	36.9 (1.1)	36.9 (1.0)	36.8 (1.2)	36.7 (1.2)	< .001
Heart rate, mean (SD), beats per min	89.0 (18.7)	89.8 (19.6)	88.5 (18.1)	88.6 (17.9)	87.3 (17.5)	86.5 (18.1)	< .001
Respiratory rate, mean (SD), breaths per min	19.2 (2.1)	19.3 (2.3)	19.1 (1.9)	19.1 (1.9)	18.9 (1.9)	18.3 (2.2)	< .001
Oxygen saturation, median (IQR), %	98.0 (3.0)	98.0 (3.0)	98.0 (3.0)	98.0 (3.0)	98.0 (3.0)	98.0 (3.0)	< .001
Systolic blood pressure, mean (SD), mmHg	135.6 (27.1)	134.0 (27.0)	136.3 (26.9)	137.6 (27.4)	138.5 (28.4)	137.7 (27.0)	< .001
Diastolic blood pressure, mean (SD), mmHg	77.7 (14.8)	77.0 (15.1)	78.1 (14.6)	78.3 (14.7)	78.4 (15.0)	78.5 (15.0)	< .001
GCS-E, *n* (%)							< .001
1	60 (0.0)	37 (0.0)	18 (0.0)	1 (0.0)	3 (0.1)	1 (0.0)	
2	184 (0.1)	127 (0.1)	43 (0.1)	8 (0.0)	4 (0.1)	2 (0.0)	
3	547 (0.3)	369 (0.4)	147 (0.2)	16 (0.1)	11 (0.2)	4 (0.0)	
4	203,073 (99.6)	84,176 (99.4)	83,653 (99.8)	21,458 (99.9)	5,304 (99.7)	8,482 (99.9)	
GCS-V, *n* (%)							< .001
1	604 (0.3)	392 (0.5)	164 (0.2)	29 (0.1)	14 (0.3)	5 (0.1)	
2	707 (0.3)	433 (0.5)	219 (0.3)	33 (0.2)	14 (0.3)	8 (0.1)	
3	384 (0.2)	229 (0.3)	125 (0.1)	22 (0.1)	7 (0.1)	1 (0.0)	
4	973 (0.5)	594 (0.7)	270 (0.3)	71 (0.3)	22 (0.4)	16 (0.2)	
5	201,196 (98.7)	83,061 (98.1)	83,083 (99.1)	21,328 (99.3)	5,265 (98.9)	8,459 (99.6)	
GCS-M, *n* (%)							< .001
1	36 (0.0)	20 (0.0)	11 (0.0)	3 (0.0)	2 (0.0)	0 (0.0)	
2	24 (0.0)	15 (0.0)	4 (0.0)	2 (0.0)	2 (0.0)	1 (0.0)	
3	108 (0.1)	67 (0.1)	35 (0.0)	4 (0.0)	2 (0.0)	0 (0.0)	
4	463 (0.2)	323 (0.4)	117 (0.1)	15 (0.1)	3 (0.1)	5 (0.1)	
5	1,418 (0.7)	886 (1.0)	407 (0.5)	76 (0.4)	27 (0.5)	22 (0.3)	
6	201,815 (99.0)	83,398 (98.5)	83,287 (99.3)	21,383 (99.5)	5,286 (99.3)	8,461 (99.7)	
Body weight, mean (SD), kg	58.7 (19.7)	57.2 (21.3)	59.6 (18.8)	60.4 (17.2)	60.6 (17.9)	58.9 (19.0)	< .001
Comorbidity	55,189 (27.1)	22,959 (27.1)	22,181 (26.4)	6,763 (31.5)	1,839 (34.6)	1,447 (17.0)	< .001
Coronary artery disease	9,432 (4.6)	3,953 (4.7)	3,687 (4.4)	1,148 (5.3)	424 (8.0)	220 (2.6)	< .001
Malignancy	23,951 (11.7)	9,979 (11.8)	9,576 (11.4)	3,021 (14.1)	742 (13.9)	633 (7.5)	< .001
Heart failure	5,261 (2.6)	2,411 (2.8)	1,984 (2.4)	563 (2.6)	200 (3.8)	103 (1.2)	< .001
COPD	2,284 (1.1)	1,116 (1.3)	805 (1.0)	248 (1.2)	61 (1.1)	54 (0.6)	< .001
Stroke	13,177 (6.5)	5,849 (6.9)	5,064 (6.0)	1,583 (7.4)	403 (7.6)	278 (3.3)	< .001
Dementia	1,913 (0.9)	983 (1.2)	635 (0.8)	207 (1.0)	52 (1.0)	36 (0.4)	< .001
Diabetes mellitus	15,125 (7.4)	6,653 (7.9)	5,841 (7.0)	1,789 (8.3)	514 (9.7)	328 (3.9)	< .001
End-stage renal disease	3,922 (1.9)	1,730 (2.0)	1,570 (1.9)	402 (1.9)	136 (2.6)	84 (1.0)	< .001
Hypertension	41,707 (20.5)	17,343 (20.5)	16,750 (20.0)	5,174 (24.1)	1,409 (26.5)	1,031 (12.1)	< .001
Liver cirrhosis	3,722 (1.8)	1,476 (1.7)	1,561 (1.9)	466 (2.2)	125 (2.3)	94 (1.1)	< .001
Peptic ulcer disease	1,634 (0.8)	708 (0.8)	605 (0.7)	211 (1.0)	70 (1.3)	40 (0.5)	< .001
Peripheral vascular disease	1,103 (0.5)	516 (0.6)	412 (0.5)	118 (0.5)	40 (0.8)	17 (0.2)	< .001
Rheumatic disease	4,232 (2.1)	1,792 (2.1)	1,743 (2.1)	485 (2.3)	128 (2.4)	84 (1.0)	< .001
Time of presentation							
Month, *n* (%)							< .001
1	18,614 (9.1)	7,375 (8.7)	7,904 (9.4)	1,908 (8.9)	470 (8.8)	957 (11.3)	
2	19,132 (9.4)	7,963 (9.4)	8,303 (9.9)	2,269 (10.6)	595 (11.2)	2 (0.0)	
3	18,656 (9.2)	7,216 (8.5)	8,461 (10.1)	2,349 (10.9)	630 (11.8)	0 (0.0)	
4	17,672 (8.7)	7,089 (8.4)	8,120 (9.7)	1,979 (9.2)	484 (9.1)	0 (0.0)	
5	17,854 (8.8)	7,387 (8.7)	7,983 (9.5)	1,997 (9.3)	487 (9.2)	0 (0.0)	
6	17,586 (8.6)	7,055 (8.3)	7,909 (9.4)	2,101 (9.8)	521 (9.8)	0 (0.0)	
7	17,398 (8.5)	7,128 (8.4)	7,809 (9.3)	2,012 (9.4)	448 (8.4)	1 (0.0)	
8	14,912 (7.3)	6,795 (8.0)	4,764 (5.7)	1,349 (6.3)	363 (6.8)	1,641 (19.3)	
9	14,306 (7.0)	6,603 (7.8)	4,731 (5.6)	1,245 (5.8)	276 (5.2)	1,451 (17.1)	
10	15,299 (7.5)	6,828 (8.1)	5,558 (6.6)	1,427 (6.6)	335 (6.3)	1,151 (13.6)	
11	15,634 (7.7)	6,675 (7.9)	5,861 (7.0)	1,329 (6.2)	323 (6.1)	1,446 (17.0)	
12	16,801 (8.2)	6,595 (7.8)	6,458 (7.7)	1,518 (7.1)	390 (7.3)	1,840 (21.7)	
Day of the week, *n* (%)							< .001
Sunday	35,195 (17.3)	14,986 (17.7)	14,571 (17.4)	3,288 (15.3)	706 (13.3)	1,644 (19.4)	
Monday	29,509 (14.5)	11,724 (13.8)	12,061 (14.4)	3,540 (16.5)	993 (18.7)	1,191 (14.0)	
Tuesday	28,080 (13.8)	11,170 (13.2)	11,835 (14.1)	3,152 (14.7)	791 (14.9)	1,132 (13.3)	
Wednesday	25,177 (12.3)	10,588 (12.5)	10,268 (12.2)	2,640 (12.3)	645 (12.1)	1,036 (12.2)	
Thursday	26,275 (12.9)	10,864 (12.8)	10,793 (12.9)	2,856 (13.3)	753 (14.1)	1,009 (11.9)	
Friday	27,665 (13.6)	11,584 (13.7)	11,333 (13.5)	2,951 (13.7)	741 (13.9)	1,056 (12.4)	
Saturday	31,963 (15.7)	13,793 (16.3)	13,000 (15.5)	3,056 (14.2)	693 (13.0)	1,421 (16.7)	
Time of the day, *n* (%)							< .001
00:00–04:00	22,736 (11.2)	9,342 (11.0)	9,937 (11.8)	1,829 (8.5)	497 (9.3)	1,131 (13.3)	
04:00–08:00	17,765 (8.7)	7,284 (8.6)	7,980 (9.5)	1,621 (7.5)	270 (5.1)	610 (7.2)	
08:00–12:00	36,784 (18.0)	15,042 (17.8)	13,798 (16.5)	4,766 (22.2)	1,790 (33.6)	1,388 (16.4)	
12:00–16:00	39,866 (19.6)	16,757 (19.8)	15,804 (18.8)	4,495 (20.9)	1,146 (21.5)	1,664 (19.6)	
16:00–20:00	43,244 (21.2)	18,247 (21.5)	17,559 (20.9)	4,700 (21.9)	932 (17.5)	1,806 (21.3)	
20:00–24:00	43,469 (21.3)	18,037 (21.3)	18,783 (22.4)	4,072 (19.0)	687 (12.9)	1,890 (22.3)	
Triage level, *n* (%)							< .001
1	114 (0.1)	82 (0.1)	23 (0.0)	7 (0.0)	2 (0.0)	0 (0.0)	
2	29,090 (14.3)	10,840 (12.8)	11,728 (14.0)	3,882 (18.1)	1,304 (24.5)	1,336 (15.7)	
3	156,049 (76.5)	62,829 (74.2)	66,409 (79.2)	16,754 (78.0)	3,855 (72.4)	6,202 (73.1)	
4	16,075 (7.9)	9,257 (10.9)	5,132 (6.1)	750 (3.5)	146 (2.7)	790 (9.3)	
5	2,536 (1.2)	1,701 (2.0)	569 (0.7)	90 (0.4)	15 (0.3)	161 (1.9)	
Waiting time, median (IQR), minute	6.4 (10.6)	5.8 (9.7)	6.3 (10.2)	8.2 (13.1)	8.0 (13.0)	10.4 (16.2)	< .001
Hospital							< .001
Academic, *n* (%)	197,337 (96.8)	80,419 (94.9)	81,875 (97.6)	21,309 (99.2)	5,245 (98.6)	8,489 (100.0)	
Community, *n* (%)	6,527 (3.2)	4,290 (5.1)	1,986 (2.4)	174 (0.8)	77 (1.4)	0 (0.0)	
Complaint							< .001
Non-trauma, *n* (%)	170,571 (83.7)	66,546 (78.6)	72,140 (86.0)	20,023 (93.2)	5,051 (94.9)	6,811 (80.2)	
Trauma, *n* (%)	33,293 (16.3)	18,163 (21.4)	11,721 (14.0)	1,460 (6.8)	271 (5.1)	1,678 (19.8)	
Outcomes							
Revisit within 72 h of the last discharge, *n* (%)	6,669 (3.3)	2,712 (3.2)	2,707 (3.2)	794 (3.7)	229 (4.3)	227 (2.7)	< .001
Mortality within 7 days after the ED visit, *n* (%)	102 (0.1)	49 (0.1)	34 (0.0)	12 (0.1)	3 (0.1)	4 (0.0)	0.601
LOS, median (IQR), minutes	128.4 (165.6)	93.2 (235.9)	120.0 (83.6)	200.5 (81.4)	319.8 (146.4)	90.7 (88.6)	< .001
Medical expenses, median (IQR), dollar	2,304.0 (2,558.0)	1,979.0 (3,082.0)	2,336.0 (2,036.0)	2,972.0 (2,495.0)	3,988.5 (4,473.5)	1,497.0 (1,037.0)	< .001

**Table 3:** Factors associated with close supervision

Variable	Multivariate logistic regression		
	Adjusted OR (95% CIs)	*p*-value	Power (95% CIs)
Age	1.009 (0.999–1.019)	0.090	
Male	1.193 (1.172–1.214)	< .001	0.99 (0.96–1.00)
Vital sign			
Body temperature	1.035 (1.025–1.044)	< .001	0.99 (0.96–1.00)
Heart rate	1.096 (1.086–1.106)	< .001	0.99 (0.96–1.00)
Respiratory rate	1.111 (1.102–1.121)	< .001	0.99 (0.96–1.00)
Oxygen saturation	0.951 (0.942–0.960)	< .001	0.99 (0.96–1.00)
Systolic blood pressure	0.967 (0.955–0.979)	< .001	0.99 (0.96–1.00)
Diastolic blood pressure	0.951 (0.940–0.963)	< .001	0.99 (0.96–1.00)
GCS-E			
1	1.157 (0.850–1.575)	0.354	
2	1.177 (0.983–1.409)	0.076	
3	1.272 (1.120–1.444)	< .001	0.93 (0.86–0.97)
4	reference		
GCS-V			
1	1.355 (1.173–1.566)	< .001	0.93 (0.86–0.97)
2	1.315 (1.144–1.511)	< .001	0.98 (0.93–1.00)
3	1.508 (1.286–1.767)	< .001	0.99 (0.96–1.00)
4	1.785 (1.613–1.975)	< .001	0.99 (0.96–1.00)
5	reference		
GCS-M			
1	1.274 (0.849–1.912)	0.242	
2	1.391 (0.881–2.195)	0.157	
3	1.156 (0.888–1.504)	0.281	
4	1.775 (1.517–2.078)	< .001	0.99 (0.964–1.00)
5	1.529 (1.362–1.718)	< .001	0.99 (0.96–1.00)
6	reference		
Body weight	0.951 (0.942–0.959)	< .001	0.99 (0.96–1.00)
Comorbidity			
Coronary artery disease	0.994 (0.955–1.034)	0.765	
Malignancy	1.134 (1.105–1.164)	< .001	0.99 (0.96–1.00)
Heart failure	1.169 (1.114–1.227)	< .001	0.99 (0.96–1.00)
COPD	1.205 (1.130–1.287)	< .001	0.99 (0.96–1.00)
Stroke	1.071 (1.035–1.108)	< .001	0.99 (0.96–1.00)
Dementia	1.017 (0.940–1.101)	0.670	
Diabetes mellitus	1.103 (1.068–1.138)	< .001	0.99 (0.964–1.00)
End-stage renal disease	1.094 (1.036–1.156)	0.001	0.94 (0.87–0.98)
Hypertension	1.029 (1.004–1.055)	0.025	0.67 (0.57–0.76)
Liver cirrhosis	0.954 (0.903–1.008)	0.096	
Peptic ulcer disease	1.051 (0.968–1.141)	0.234	
Peripheral vascular disease	1.143 (1.039–1.257)	0.006	0.78 (0.69–0.86)
Rheumatic disease	1.065 (1.012–1.122)	0.017	0.58 (0.48–0.68)
Time of presentation			
Month			
1	reference		
2	0.95 (0.915–0.986)	0.007	0.81 (0.72–0.88)
3	0.861 (0.829–0.894)	< .001	0.99 (0.96–1.00)
4	0.922 (0.887–0.958)	< .001	0.99 (0.96–1.00)
5	0.969 (0.933–1.006)	0.103	
6	0.887 (0.854–0.922)	< .001	0.99 (0.96–1.00)
7	0.975 (0.939–1.013)	0.201	
8	1.513 (1.453–1.576)	< .001	0.99 (0.96–1.00)
9	1.509 (1.448–1.572)	< .001	0.99 (0.96–1.00)
10	1.343 (1.290–1.398)	< .001	0.99 (0.96–1.00)
11	1.240 (1.191–1.290)	< .001	0.99 (0.96–1.00)
12	1.146 (1.102–1.192)	< .001	0.99 (0.96–1.00)
Day of the week			
Sunday	reference		
Monday	0.899 (0.873–0.926)	< .001	0.99 (0.96–1.00)
Tuesday	0.911 (0.885–0.938)	< .001	0.99 (0.96–1.00)
Wednesday	1.030 (0.999–1.061)	0.057	
Thursday	0.976 (0.947–1.005)	0.105	
Friday	1.003 (0.973–1.033)	0.867	
Saturday	1.032 (1.003–1.063)	0.030	0.59 (0.49–0.69)
Time of the day			
00:00–04:00	reference		
04:00–08:00	0.989 (0.953–1.026)	0.553	
08:00–12:00	0.937 (0.908–0.967)	< .001	0.99 (0.95–1.00)
12:00–16:00	0.984 (0.954–1.015)	0.309	
16:00–20:00	0.955 (0.927–0.985)	0.004	0.88 (0.80–0.94)
20:00–24:00	0.921 (0.893–0.950)	< .001	0.99 (0.96–1.00)
Triage level			
1	1.393 (1.128–1.721)	0.002	0.89 (0.81–0.94)
2	1.033 (1.011–1.056)	0.003	0.87 (0.79–0.93)
3	reference		
4	1.786 (1.726–1.848)	< .001	0.99 (0.96–1.00)
5	3.187 (2.914–3.486)	< .001	0.99 (0.96–1.00)
Waiting time	0.963 (0.955–0.972)	< .001	0.99 (0.96–1.00)
Hospital			
Academic	reference		
Community	2.004 (1.910–2.104)	< .001	0.99 (0.96–1.00)
Complaint			
Non-trauma	reference		
Trauma	1.521 (1.484–1.559)	< .001	0.99 (0.96–1.00)

Comorbidities such as malignancy, heart failure, COPD, stroke, diabetes mellitus, and end-stage renal disease were also associated with increased odds of close supervision (*p* < 0.001 for all). Interestingly, patients presenting in community hospitals were twice as likely to be closely supervised compared to those in academic hospitals (OR = 2.004, *p* < 0.001). These findings suggest that a variety of factors, including patient demographics, vital signs, comorbidities, and the type of hospital, can influence the level of supervision provided in emergency settings.

### Main results

In the multivariable analysis, a longer independent practice time was associated with higher revisit ORs even when other factors such as age, gender, and underlying medical conditions were controlled for (Appendix 1). Factors such as older age, male sex, higher body temperature, higher heart rate, lower systolic blood pressure, and certain underlying medical conditions (such as cancer, heart failure, stroke, diabetes mellitus, end-stage renal disease, hypertension, liver cirrhosis, and rheumatic disease) were found to be independent predictors of an ED revisit within 72 h.

Patients who presented to the ED in the months of May, June, August, September, October, or November were found to have an increased risk of revisiting the ED within 72 h. Patients who presented to the ED on Thursdays, Fridays, or Saturdays also had an increased risk of revisiting the ED within 72 h. Furthermore, patients treated by a resident who practiced independently for more than 120 min had a higher OR for revisiting the ED within 72 h than those patients treated by a resident who practiced independently for less than 30 min, even when other factors were controlled for (Table 4).

**Table 4:** Factors associated with revisiting the ED within 72 h for patients discharged from the ED

Variable	Multivariate logistic regression		
	Adjusted OR (95% CIs)	*p*-value	Power (95% CIs)
Age	1.355 (1.311–1.401)	< .001	0.99 (0.96–1.00)
Male	1.163 (1.106–1.223)	< .001	0.99 (0.96–1.00)
Vital sign			
Body temperature	1.043 (1.006–1.081)	0.021	0.66 (0.56–0.75)
Heart rate	1.225 (1.191–1.260)	< .001	0.99 (0.96–1.00)
Respiratory rate	1.023 (0.995–1.052)	0.110	
Oxygen saturation	-	-	
Systolic blood pressure	0.938 (0.904–0.973)	0.001	0.94 (0.87–0.98)
Diastolic blood pressure	1.004 (0.968–1.041)	0.845	
GCS-E			
1	0.812 (0.257–2.560)	0.722	
2	0.847 (0.406–1.768)	0.659	
3	1.331 (0.893–1.984)	0.160	
4	reference		
GCS-V			
1	0.932 (0.571–1.521)	0.779	
2	1.276 (0.828–1.968)	0.269	
3	1.143 (0.685–1.906)	0.610	
4	0.918 (0.663–1.272)	0.609	
5	reference		
GCS-M			
1	3.103 (1.057–9.107)	0.039	0.50 (0.40–0.60)
2	2.730 (0.774–9.626)	0.118	
3	1.001 (0.428–2.339)	0.998	
4	0.734 (0.420–1.282)	0.277	
5	0.873 (0.599–1.274)	0.482	
6	reference		
Body weight	-	-	
Comorbidity			
Coronary artery disease	1.059 (0.956–1.172)	0.272	
Malignancy	1.574 (1.470–1.687)	< .001	0.99 (0.964–1.00)
Heart failure	1.162 (1.027–1.315)	0.018	0.58 (0.48–0.68)
COPD	0.943 (0.790–1.125)	0.512	
Stroke	1.198 (1.098–1.306)	< .001	0.99 (0.946–1.00)
Dementia	0.890 (0.720–1.100)	0.280	
Diabetes mellitus	1.131 (1.042–1.227)	0.003	0.84 (0.75–0.91)
End-stage renal disease	1.429 (1.251–1.632)	< .001	0.99 (0.964–1.00)
Hypertension	1.174 (1.095–1.259)	< .001	0.99 (0.964–1.00)
Liver cirrhosis	1.682 (1.493–1.896)	< .001	0.99 (0.964–1.00)
Peptic ulcer disease	1.123 (0.920–1.370)	0.254	
Peripheral vascular disease	1.216 (0.966–1.531)	0.097	
Rheumatic disease	1.177 (1.025–1.351)	0.021	0.60 (0.50–0.70)
Time of presentation			
Month			
1	reference		
2	1.091 (0.969–1.229)	0.148	
3	1.041 (0.923–1.174)	0.515	
4	1.116 (0.990–1.259)	0.073	
5	1.157 (1.027–1.304)	0.016	0.67 (0.57–0.76)
6	1.239 (1.101–1.393)	< .001	0.94 (0.87–0.98)
7	1.112 (0.985–1.255)	0.085	
8	1.192 (1.052–1.350)	0.006	0.72 (0.62–0.81)
9	1.191 (1.050–1.351)	0.006	0.74 (0.64–0.82)
10	1.203 (1.064–1.361)	0.003	0.81 (0.72–0.88)
11	1.156 (1.022–1.307)	0.021	0.68 (0.58–0.77)
12	1.108 (0.980–1.252)	0.102	
Day of the week			
Sunday	reference		
Monday	1.061 (0.970–1.161)	0.195	
Tuesday	1.044 (0.953–1.144)	0.358	
Wednesday	1.062 (0.966–1.166)	0.213	
Thursday	1.109 (1.011–1.216)	0.028	0.59 (0.49–0.69)
Friday	1.144 (1.046–1.252)	0.003	0.76 (0.66–0.84)
Saturday	1.109 (1.016–1.211)	0.021	0.68 (0.58–0.77)
Time of the day			
00:00–04:00	reference		
04:00–08:00	0.957 (0.861–1.063)	0.409	
08:00–12:00	0.764 (0.697–0.838)	< .001	0.99 (0.96–1.00)
12:00–16:00	0.780 (0.712–0.856)	< .001	0.99 (0.96–1.00)
16:00–20:00	0.805 (0.735–0.882)	< .001	0.99 (0.95–1.00)
20:00–24:00	0.814 (0.744–0.892)	< .001	0.99 (0.96–1.00)
Triage level			
1	1.666 (0.815–3.408)	0.162	
2	1.057 (0.988–1.132)	0.109	
3	reference		
4	0.966 (0.870–1.072)	0.515	
5	1.262 (1.005–1.584)	0.045	0.47 (0.37–0.57)
Waiting time	1.011 (0.986–1.036)	0.392	
Hospital			
Academic	reference		
Community	1.771 (1.566–2.002)	< .001	0.99 (0.96–1.00)
Complaint			
Non-trauma	reference		
Trauma	0.607 (0.553–0.666)	< .001	0.99 (0.96–1.00)
Resident independent practice time, minutes			
< 30	reference		
30 to 120	1.034 (0.978–1.093)	0.239	
120 to 210	1.113 (1.025–1.208)	0.011	0.70 (0.65–0.74)
> 210	1.259 (1.094–1.449)	0.001	0.87 (0.79–0.93)
Non-recorded	1.042 (0.904–1.201)	0.570	

The regression analyses of secondary outcomes revealed that patients who were treated by residents under close supervision were associated with the highest mortality OR, as well as a longer length of stay and higher cost than those patients who were practiced independently by residents within 30–210 min (Table 5).

**Table 5:** Regression analyses of secondary outcomes

Variable	Mortality within 7 days after the ED visit	LOS	Medical expenses
	Adjusted OR (95% CI)	Adjusted beta coefficients (95% CI)	Adjusted beta coefficients (95% CI)
Resident independent practice time, minutes			
< 30	reference	reference	reference
30 to 120	0.674* (0.592–0.767)	-173.295* (-183.034 to -163.556)	-638.489* (-690.164 to -586.814)
120 to 210	0.259* (0.178–0.376)	-178.340* (-193.635 to -163.046)	-183.385* (-264.540 to -102.230)
> 210	0.318* (0.173–0.584)	-80.715* (-108.753 to -52.677)	1314.678* (1165.921 to 1463.435)
Non-recorded	0.273* (0.113–0.662)	-320.863* (-344.008 to -297.717)	-2013.822* (-2136.709 to -1890.935)

^*^Indicates *p*-value < 0.05

## Discussion

We noted a number of factors associated with less resident autonomy provided by attending physicians, including being in the first few months of the academic year and having patients with the highest or lowest emergency index. In this study, we obtained some evidence to determine that initial supervision of up to 120 min when a resident sees some ED patients provides sufficient resident autonomy without increasing the ED revisit rate. At the same time, an independent practice time of 30 to 120 min did not increase mortality, LOS, or medical costs. The results indicate that an independent practice time of within 30–120 min is associated with a non-inferior odds of revisiting the ED within 72 h and no higher 7-day mortality than an independent practice time of < 30 min. Furthermore, there was a statistically significant association between an independent practice time > 120 min and an increased odds of revisiting the ED and higher beta-coefficients for total medical expenses. These results suggest that providing residents and PGYs with an appropriate level of autonomy while ensuring adequate supervision by attending physicians in the ED can lead to preferred patient outcomes without negative consequences.

**Fig. 4:**
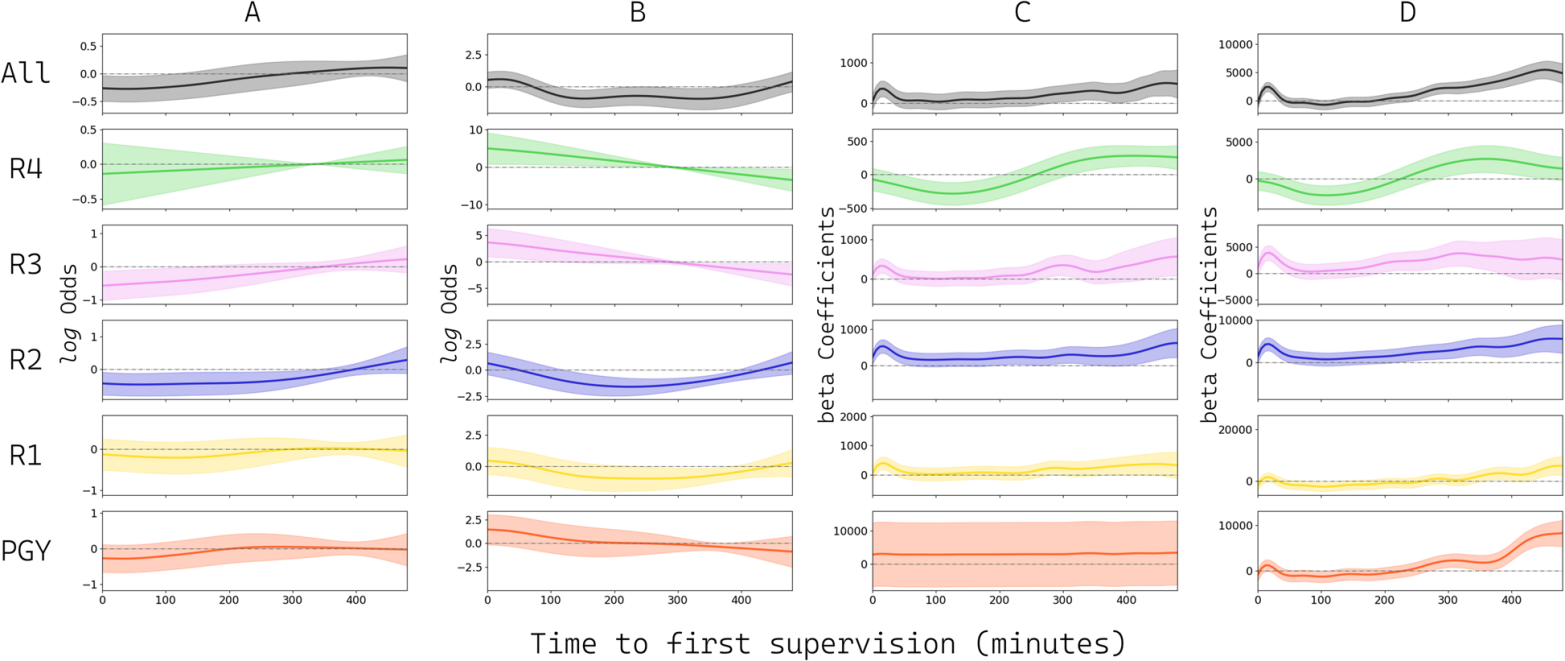
Results of the adjusted analysis using aGAM of the time to first supervision and its relationship to patient outcomes according to the resident level. **A** Revisiting the ED within 72 hours. The trend with regard to resident experience level showed that as the residents gained more experience (PGY, R1, R2), the acceptable duration of supervision increased, with a negative log odds. **B** Mortality in sevendays after the ED visit. **C** LOS in the ED. **D** Total medical expenses during the ED visit. There was a noticeable peak within the first 60 minutes in the LOS and cost, while the first 100 minutes was associated with a higher log odds-on mortality. This may indicate that patients in poor condition requiring more care received care under supervision by an attending physician earlier, which could have been initiated by either the attending physician or the resident calling for help

The degree of residency autonomy varies by situation, and Jenkin’s research demonstrated this difference between community and university hospitals [33]. In general, the condition of patients in university hospitals may be more complex, and patient safety issues may be more prominent, which limits the attainment of autonomy. Providing an environment for residents to practice independently requires proper planning. EPAs are proposed to provide autonomy based on the proficiency of employees [4, 19]. Jenkin indicated that teamwork and collective problem-solving provide interns with sufficient decision-making ownership while still allowing their superiors to fine-tune their decisions if necessary [33]. In the EDs in this study, the attending physicians are legally responsible for the patients, and the residents and PGYs are treated as physician learners who share the workload of the attending physicians and learn through practice. The patients in this study in the university hospital ED were divided into two areas during triage. Critically ill patients could be treated immediately by senior residents and attending physicians, and relatively non-critical patients could be independently treated by resident physicians first, followed by the supervision of attending physicians. In the results, we noted that attending physicians delegated approximately two-thirds of patient care to residents and PGYs, and 45% of these patients (116,742 out of 258,738 academic ED patients) had close supervision while being treated by a resident. In contrast, 65% of patients (5,588 out of 8,643 community ED patients) were treated under close supervision by an attending physician in the community hospital. In general, this strategy allows attending physicians to intervene early in patient management and should reduce patient safety concerns, while it may increase attending physician tension and reduce their productivity. In addition, such close supervision may interrupt residents' clinical thinking and treatment decisions, reducing autonomy. Close supervision did not yield lower mortality rates in the results, which may be due to more severe or critical conditions that residents identified earlier and sought early help from their attending physicians. Only some patients who were treated by a resident practicing independently for more than 400 min had an increased risk of mortality (Fig. 4). Those who were sicker may have been admitted rather than discharged, which may have interfered with the analysis of revisits, for which we only included those patients who were discharged. Approximately 42% of patients (84,709 of 203,864 discharged patients) were treated by a resident who was closely supervised, with no statistically significant difference in revisit rates compared with those treated by residents practicing independently for 30 to 120 min. According to different levels of residents, attending physicians may be able to give more experienced residents more time to practice independently (Fig. 4). This trust may be associated with less close supervision in the middle and later parts of the academic year (Fig. 3).

The role of residents in patient care is crucial, and the level of autonomy should reflect their level of training, the supervising physician's confidence in their skills, and the complexity of the patient's medical situation. As a general rule, residents work under the guidance of an experienced attending physician. However, as they gain experience and knowledge, they may be given more independence in making decisions and managing patient care. The result of this study revealed that supervision occurred earlier during the initial months of the academic year and that there may be potential for increased autonomy as residents progress from PGY to R1 to R2 levels (Fig. 4), which aligns with the finding of previous research on the topic [9]. It is essential for residents to be able to develop their clinical skills and judgment gradually through increased autonomy, while still receiving support and guidance from more seasoned physicians. The optimal timing of independent practice in the ED can vary depending on various factors, including the resident's level of training, the complexity of the patient's condition, and the attending physician's comfort level with the resident's abilities. The attending physician might provide close supervision during the early stages of the resident's training and for complex cases. As the resident gains experience and knowledge, he or she may be given more independence, but the attending physician should still be available for guidance and support as needed.

### Limitations

This study has limitations, one of which is the lack of documentation, which may have affected the accuracy of the data regarding the independent practice time among residents. This could be due to the fast-paced and demanding environment in the ED, leading to retroactive documentation or oral orders being recorded later when there was more time. This issue can be seen from the fact that the group with missing documentation had the shortest median LOS compared with the other groups (Table 2). This is a common challenge in emergency medicine and highlights the need for further research and solutions to enhance the documentation process in EDs.

An additional source of potential bias in our study was the presence of typos, arising from human errors during data entry. In the dataset encompassing all subjects (*n* = 258,738), a total of 1074 (0.4%) cases exhibited such errors. These cases were subsequently excluded from the analysis to maintain data reliability. Upon examination, the distribution of these typos across different groups, based on the duration of residents' independent practice, was not found to be statistically significant (*p*-value = 0.100) (Table 6). Thus, the exclusion of these cases might not introduce bias into our results.

**Table 6:** Proportional distribution of subjects exhibiting data entry errors (‘typos’) across various durations of resident independent practice time

Variable	All subjects (*n* = 258,738)	Resident independent practice time (minutes)					
		< 30	30 to 120	120 to 210	> 210	Non-recorded	*p*-value
Study population (%)	258,738 (99.6)	116,742	101,179	25,498	6,640	8,679	0.100
Typo (%)	1074 (0.4)	479	402	115	35	43	

Furthermore, the study only included data from three urban hospitals, and further studies are needed to replicate these findings and to examine the effect of supervision on other quality and safety indicators in the ED.

Another limitation is that in a retrospective study, it is hard to determine whether active supervision was initiated by the attending physician or was due to a resident's call for help. The association of a shorter independent practice time with increased mortality may result from residents calling for help early when a patient’s condition is concerning. Furthermore, patient safety and medical quality issues can be measured by indicators other than the revisit rate, mortality rate, and LOS. It is important to note that the level of independence given to residents can vary based on various factors. These results should be considered in the context of these factors and may not be applicable to all situations. For future studies, we recommend a more in-depth exploration of the factors that contribute to the optimal range of independent practice time for residents. This could include investigating the impact of different supervision styles, the complexity of cases handled by residents, and the influence of specific training programs or educational interventions. Additionally, longitudinal studies could provide valuable insights into the long-term effects of different levels of resident autonomy on patient outcomes and the professional development of the residents themselves.

## Conclusion

This study suggests that the appropriate level of autonomy for residents in the ED may be an independent practice time of 30–120 min. A longer independent practice time was associated with negative outcomes such as higher revisit and mortality rates. In addition, the results from the analysis revealed that there might be an opportunity for increased autonomy for residents as they progress from PGY to R1 to R2 levels. Overall, it is important to strike a balance between providing sufficient supervision to ensure patient safety and allowing residents to gain the experience and independence they need to become fully licensed physicians while considering individual patient characteristics and disease factors.

### Supplementary Information

**Additional file 1:** **Appendix 1.** Stepwise multivariate regression analysis of resident independent practice time and 72-hour ED revisits, with grouping of similar odds ratios. **Appendix 2.** Comparison of vital signs and glasgow coma scale scores before and after imputation for missing data in a clinical dataset.

## Data Availability

The data that support the findings of this study are available on request from the corresponding author, YP Chen and CH Huang. The data is also available through application to the NTUH-iMD.
