# Supplementary material for: Association between independent practice time and patient outcomes in the emergency department: a retrospective study of residents in three urban hospitals in Taiwan
Source: BMC Emerg Med. 2023 Sep 7;23:103. doi: 10.1186/s12873-023-00877-9 (PMC10483807; doi:10.1186/s12873-023-00877-9)
Supplement: Supplementary file 1 — Additional file 1: Appendix 1. Stepwise multivariate regression analysis of resident independent practice time and 72-hour ED revisits, with grouping of similar odds ratios. Appendix 2. Comparison of vital signs and glasgow coma scale scores before and after imputation for missing data in a clinical dataset. [file 12873_2023_877_MOESM1_ESM.docx]

Appendix 1 Stepwise multivariate regression analysis of resident independent practice time and 72-hour ED revisits, with grouping of similar odds ratios.

| Resident independent practice time, minutes | Case number, n (%) | Adjusted OR  (95% CI) | p-value | Adjusted OR  (95% CI) | p-value | Adjusted OR  (95% CI) | p-value |
| --- | --- | --- | --- | --- | --- | --- | --- |
| < 30 | 84,709 (43.4) | reference | | reference | | reference | |
| 30 to 60 | 33,258 (17.0) | 1.018 (0.946-1.096) | 0.627 | 1.018 (0.946-1.096) | 0.627 | 1.034 (0.978-1.093) | 0.239 |
| 60 to 90 | 30,298 (15.5) | 1.040 (0.965-1.122) | 0.307 | 1.043 (0.979-1.112) | 0.191 |  |  |
| 90 to 120 | 20,305 (10.4) | 1.048 (0.961-1.143) | 0.291 |  |  |  |  |
| 120 to 150 | 11,706 (6.0) | 1.093 (0.983-1.215) | 0.102 | **1.097 (1.004-1.199)** | **0.041*** | **1.113 (1.025-1.208)** | **0.011*** |
| 150 to 180 | 6,264 (3.2) | 1.106 (0.963-1.270) | 0.155 |  |  |  |  |
| 180 to 210 | **3,513 (1.8)** | **1.195 (1.003-1.424)** | **0.047*** | **1.195 (1.003-1.424)** | **0.047*** |  |  |
| 210 to 240 | 2,042 (1.1) | 1.233 (0.985-1.542) | 0.067 | **1.229 (1.030-1.466)** | **0.022*** | **1.259 (1.094-1.449)** | **0.001*** |
| 240 to 270 | 1,259 (0.6) | 1.223 (0.928-1.612) | 0.153 |  |  |  |  |
| >270 | **2,021 (1.0)** | **1.314 (1.057-1.634)** | **0.014*** | **1.314 (1.057-1.634)** | **0.014*** |  |  |

Appendix 2 Comparison of vital signs and glasgow coma scale scores before and after imputation for missing data in a clinical dataset.

| **Variable** | **Missing value** | **Raw data** | | **Post imputation** | |
| --- | --- | --- | --- | --- | --- |
|  | **Number (%)** | **mean (SD)** | **median (IQR)** | **mean (SD)** | **median (IQR)** |
| **Body temperature, °C** | 119 (0.05) | 37 (0.8) | 36.8 (0.8) | 36.9 (1.1) | 36.8 (0.8) |
| **Heart rate, beats per min** | 396 (0.15) | 90.7 (19.2) | 89.0 (26.0) | 90.5 (19.5) | 89.0 (26.0) |
| **Respiratory rate, breaths per min** | 240 (0.09) | 19.3 (2.2) | 20.0 (2.0) | 19.3 (2.2) | 20.0 (2.0) |
| **Oxygen saturation, %** | 528 (0.20) | 97.3 (2.0) | 98.0 (3.0) | 97.1 (4.8) | 98.0 (3.0) |
| **Systolic blood pressure, mmHg** | 776 (0.30) | 135.8 (26.4) | 132.0 (34.0) | 135.4 (27.4) | 132.0 (33.0) |
| **Diastolic blood pressure , mmHg** | 809 (0.31) | 77.6 (14.4) | 77.0 (18.0) | 77.3 (15.0) | 77.0 (18.0) |
| **GCS-E** | 0 (0.00) | 4.0 (0.2) | 4.0 (0.0) | 4.0 (0.2) | 4.0 (0.0) |
| **GCS-V** | 1,371 (0.53) | 4.9 (0.5) | 5.0 (0.0) | 4.9 (0.5) | 5.0 (0.0) |
| **GCS-M** | 0 (0.00) | 6.0 (0.3) | 6.0 (0.0) | 6.0 (0.3) | 6.0 (0.0) |
| **Body weight, kg** | 11,226 (4.34) | 61.2 (15.7) | 60.0 (18.0) | 58.5 (19.8) | 59.7 (20.0) |
